# Recent Advancements in Aptamer-Based Surface Plasmon Resonance Biosensing Strategies

**DOI:** 10.3390/bios11070233

**Published:** 2021-07-10

**Authors:** Chia-Chen Chang

**Affiliations:** 1 Department of Medical Biotechnology and Laboratory Science, Chang Gung University, Taoyuan 333, Taiwan; chang@mail.cgu.edu.tw; Tel.: +886-3-2118800 (ext. 5086); 2 Kidney Research Center, Department of Nephrology, Chang Gung Memorial Hospital, Taoyuan 333, Taiwan

**Keywords:** surface plasmon resonance, aptamer, one-site binding, two-site binding, aptasensor

## Abstract

Surface plasmon resonance (SPR) can track molecular interactions in real time, and is a powerful as well as widely used biological and chemical sensing technique. Among the different SPR-based sensing applications, aptamer-based SPR biosensors have attracted significant attention because of their simplicity, feasibility, and low cost for target detection. Continuous developments in SPR aptasensing research have led to the emergence of abundant technical and design concepts. To understand the recent advances in SPR for biosensing, this paper reviews SPR-based research from the last seven years based on different sensing-type strategies and sub-directions. The characteristics of various SPR-based applications are introduced. We hope that this review will guide the development of SPR aptamer sensors for healthcare.

## 1. Introduction

Biosensors are critical analytical devices not only in biochemical and molecular diagnostics, but also in food analysis and environmental monitoring. Numerous labeling strategies have been developed via the covalent attachment of chemical tags to molecules to quantitatively assess the binding events among biomolecules. However, the use of tags may cause steric hindrance, resulting in a change in the binding capacities of molecules for their targets. In addition, labeling molecules for large-scale studies is complicated, tedious, and limited by various factors. Conversely, label-free bioanalysis technologies eliminate labeling or tagging procedures, thus allowing the use of natural biomolecules that are suitable for numerous applications in the biomedical field [1,2,3,4]. Various label-free analytical techniques have been reported, involving the use of the quartz crystal microbalance [5], biolayer interferometry [6], surface acoustic waves [7], silicon nanowires [8], and surface plasmon resonance (SPR) [9]. Among them, there has been significant interest in biosensors based on SPR owing to their inherently attractive features, such as rapid detection in real time and the possibility of on-chip integration for smart sensor systems [10,11,12]. SPR-based biosensing is a powerful analytical technique for tracking molecular interactions, and makes molecule detection simple and rapid. There has been much research based on the classical Kretschmann configuration of SPR [13,14,15]. SPR is characterized by an evanescent wave field, which is generated across an interface by total internal reflection and propagates at ~100 nm from the surface of a gold film [16]. Hence, an SPR sensor can detect binding events that occur at the gold surface, resulting in changes in the refractive index and shifts in the SPR resonance angle. Optical transduction directly converts a molecular binding event or a biological reaction into a physically measurable signal, which is proportional to the concentration of the analyte molecules. Label-free SPR detection provides an efficient method, and the use of nanomaterials as a label improves the SPR response signals. Although SPR belongs to label-free techniques in terms of signal generation mechanisms, SPR detection using nanomaterials for the signal enhancement can be considered as a ‘label-based’ strategy [17]. Due to these properties, SPR has been widely used as a sensing technique in food safety [18,19,20], medical diagnostics [21,22,23,24], and environmental monitoring [25,26,27]. Typically, an antibody as the bio-recognition element is immobilized onto the gold surface, leading to the selective interaction of target proteins or biomolecules and the generation of SPR signals. However, the high cost, extensive purification steps, low-temperature storage, and inherent batch-to-batch variation are significant challenges inhibiting the use of antibodies as bio-receptors [28,29,30].

The success of antibody-based SPR detection has inspired research in the field of aptamers. Recently, novel combinatorial nucleic acid and peptide molecules have attracted the attention of researchers working in various areas of sensing, ranging from molecular diagnosis to analytical chemistry [31,32,33,34]. These molecules are referred to as aptamers, and have been proposed as alternatives to antibodies. For example, nucleic acid aptamers are small sequences of DNA or RNA that fold into well-defined and stable 3D sequence-dependent structures. This inherent feature enables them to efficiently interact with molecular targets, ranging from metal ions and small organic compounds to large protein targets or even complex molecules [35,36]. Aptamers are successfully generated from combinatorial nucleic acid libraries using in vitro selection methods, which are usually more cost-effective than antibody production and purification. In addition to the aforementioned advantages of aptamers, nucleic acid aptamers, in particular, provide distinct advantages. For example, they can be chemically modified with tags (such as a fluorescent molecules or gold particles) or integrated into nucleic acid nanostructures without any negative impact on the binding affinity toward their targets [37]. Moreover, unlike protein-based antibodies and nanobodies, the denaturation of nucleic acid aptamers under unfavorable conditions is reversible [38]. Although RNA aptamers were identified first, DNA aptamers have been extensively employed in sensors because of their resistance to base-catalyzed hydrolysis [39]. Consequently, by incorporating these nucleic acid aptamers into various sensing platforms, it is possible to subject these probes to repeated use without loss of functionality, thereby allowing a device to be potentially recyclable. Numerous aptamer-based approaches have been reported for chemical and biological detection, such as surface-enhanced Raman spectroscopy [40,41], SPR [42,43], fluorescence [44,45], colorimetry [46,47], and electrochemistry [48,49].

**Figure 1:**
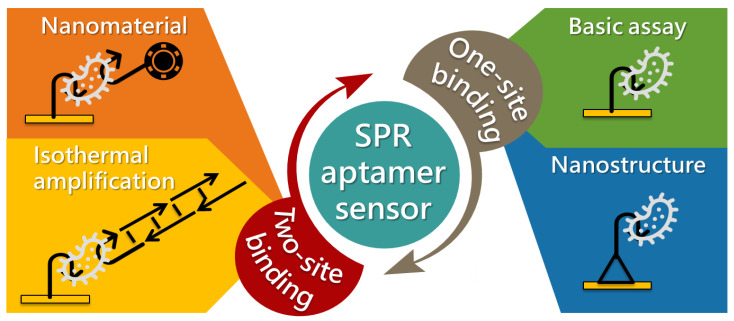
Summary of different types of aptamer-based SPR sensors.

These aptamer-based SPR methods have been extensively used owing to their simplicity. In this review, we describe SPR sensors that use aptamers as sensing materials for biological and chemical detection applications. Many review articles published by experts in SPR have highlighted the advantages of the working principles, setups, and applications [50,51,52,53,54]. There are many more reviews on the improvement of the SPR sensitivity, particularly for integration with other techniques, such as those using nanomaterials [55,56,57,58]. Comparatively fewer reviews exist on aptamer-based SPR sensors [59]. For this reason, we focus mainly on articles published in the last seven years (2014–2020). Considering the large number and variety of aptasensors reported, this review focuses mainly on two detection methods: direct (one-site binding) and sandwich (two-site binding) (Figure 1). Finally, future challenges and perspectives on the development of SPR aptasensors are described.

## 2. Direct (One-Site Binding) Detection Mode

### 2.1. Basic SPR Assay

**Figure 2:**
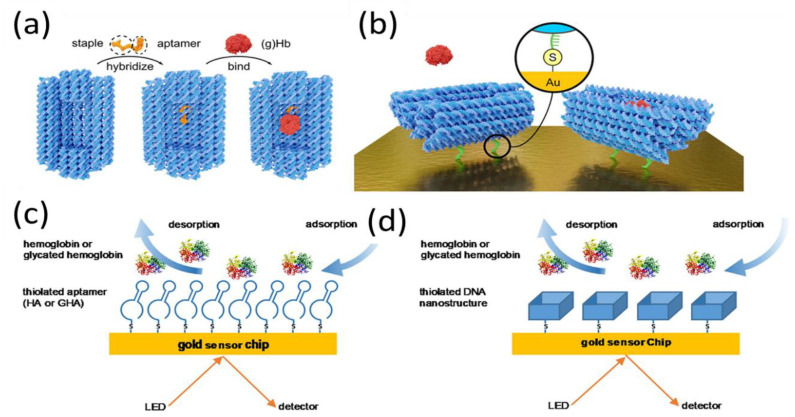
(**a**) Aptamers embedded into the DNA origami cage by hybridization, with glycated Hb (gHb) subsequently interacting with the aptamers. (**b**) Aptamer-modified DNA origami cage immobilized on a gold chip and bonded to gHb. Inset: DNA cage with thiol-modified ssDNA strands covalently bound to the gold surface. SPR assays for (**c**) thiolated aptamer and (**d**) thiolated aptamer-embedded DNA nano-cage. Reproduced with permission from [73]. Elsevier B.V., 2020.

The basic sensing mechanism of SPR aptasensors is similar to that of other sensors: aptamers are immobilized on the sensing surface, following which the aptamer probe recognizes and interacts with its target; in the last step, the optical transducer converts this interaction information into detectable signals. This direct detection approach is much faster than other SPR sensing modes, as fewer steps and less time are required. Notably, most small molecules bind aptamers only with the one-site binding configuration because there is no room for the aptamer to interact with a second molecule [60]. Therefore, many SPR aptasensors have been developed based on this direct strategy [61,62,63,64,65]. For example, Wu et al. and Ashley et al. immobilized a biotin-modified aptamer probe on an avidin-modified chip through streptavidin–biotin interaction for the detection of aflatoxin in vinegar and lysozyme in milk [66,67]. RNA aptamers were anchored on the sensor surface via thiol–gold interactions to evaluate the binding kinetics of various molecules [68,69,70]. However, as SPR is sensitive to changes in the refractive index at the sensor surface caused by the mass of the binding component, it is challenging to achieve satisfactory sensor performance in the detection of small molecules [71,72]. Duanghathaipornsuk et al. believed that the strength of the binding affinity also affects the sensing performance. To improve the binding stability and strength of aptamers to target proteins, DNA nanocages were fabricated for the SPR sensing of hemoglobin and glycated hemoglobin (Figure 2) [73]. The 3D DNA cage contained two selected, closed cavities with aptamers designed to fit, capture, and enhance binding and selectivity to target proteins. Compared to the single-stranded DNA aptamer, the DNA aptamer-embedded origami cage structure yielded 22-fold and 9-fold enhancements of binding affinity and selectivity toward glycated hemoglobin respectively, rendering it a promising tool for the enhancement of SPR performance.

**Figure 3:**
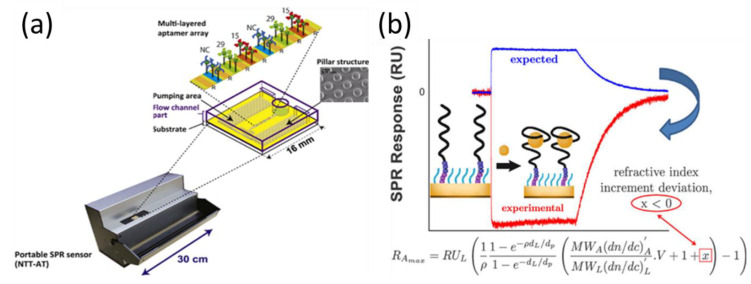
(**a**) Portable SPR measurement setup with a capillary-driven aptamer array-based flow chip. (**b**) Experimental and expected SPR curves after the addition of tyrosinamide. Reproduced with permission from [78,82]. Elsevier B.V., 2016. American Chemical Society, 2021.

Multiplexed molecular sensing systems aim to detect multiple targets simultaneously from a single sample, thereby reducing the required volume and minimizing the detection time [74,75,76]. There has been common interest in the development of analytical tools for biomolecule detection using SPR sensors. Chen et al. constructed a four-chambered microfluidic SPR system based on microarrays of RNA aptamers for the detection of human thrombin and vascular endothelial growth factor (VEGF) proteins [77]. In the microfluidic format, RNA aptamers can be produced directly and quickly by the surface transcription reaction of T7 RNA polymerase, thus allowing for one-step multiplexed protein biosensing. Although a single SPR aptamer array permits the simultaneous analysis of multiple target molecules, the reliability of SPR chips is significantly affected by the reproducibility of the sensor array. To overcome this limitation, Inoue et al. reported an SPR aptamer array using an inkjet spotter that could precisely control the position and volume of an ejected aptamer solution [78]. They used a portable multi-analysis SPR device with a capillary-driven flow chip for thrombin detection (Figure 3a). SPR signals arising from different concentrations of thrombin were observed simultaneously. Based on this method, the reproducibility of SPR arrays was significantly improved by minimizing the manual intervention in the preparation process and by using the BlockAce reagent, which is widely employed as a blocking solution with ELISA technology for separating biomolecule spots. Based on this method, the detection limit of the SPR aptamer array was comparable to that of other SPR sensors (1 nM). Non-specific attachment in SPR arrays has often been a critical problem that affects the reliability of assay results. To this end, Duanghathaipornsuk et al. reported that the inclusion of 3,6-dioxa-8-mercaptooctan-1-ol (DMOL) in a self-assembled monolayer-modified array surface can mitigate the nonspecific binding of proteins and impart more degrees of freedom to the aptamers for interacting with the targets [79]. In addition to sensing applications, SPR arrays can be used to assess the surface density of aptamer strands. Gyurcsányi et al. reported that an optimal surface density of the aptamers yielded the best measured affinity, which is largely determined by the size of the target [80].

In general, the SPR signal, which is proportional to the molecular weight and refractive index increment, is expected to be positive and increase with the amount of target molecules [71,81]. However, nonconventional SPR signals were observed in the detection of tyrosinase by Bonnet et al. (Figure 3b), who reported that the observation of negative SPR signals during analyte recognition resulted from the conformational transition of aptamers [82]. They found that configuration rearrangement of the aptamer resulted in a deviation of the refractive index increment of a small molecule/aptamer complex from the sum of the refractive index increments of the individual entities. These results provide new ideas and insights for understanding the effects of the nonlinearity of the refractive index increment on SPR signal changes.

Typically, direct-mode-based SPR aptasensors for biomolecules are achieved at nanomolar concentrations. Consequently, there has been considerable effort to further amplify the SPR detection response, such as in terms of nanomaterial or enzyme amplification, the details of which are introduced in the next section.

### 2.2. SPR Assay Using Nanostructural Surface Design

Direct assays are less flexible, and there is no secondary probe to amplify the SPR signal. Another major contribution to the development of direct detection-based SPR aptasensors was from the use of nanomaterials and conducting metal oxides as aptamer probe-immobilized substrates to enhance the SPR performance. Chang et al. fabricated an Au/ZnO nanocomposite that allowed for a sensitivity higher than that of standard gold-based substrates [83]. By combining DNA aptamers, this nanocomposite has also been applied for environmental monitoring [84]. Recently, graphene-related materials have been explored as coatings of SPR chips with the aim of enhancing the SPR sensor performance [85,86,87]. With regard to the application of graphene-related materials to SPR sensors, Wu et al. reported that a graphene-on-gold SPR sensor can be more sensitive than the conventional configuration of the SPR chip, owing to the optical properties of graphene and increased adsorption of biomolecules [88]. They found that the highest sensitivity was achieved with a single layer of graphene, which was five times more sensitive than the conventional gold thin-film SPR biosensor. However, a graphene thickness exceeding 10 nm substantially restricts optical absorption, resulting in a decrease in the SPR sensitivity [89].

**Figure 4:**
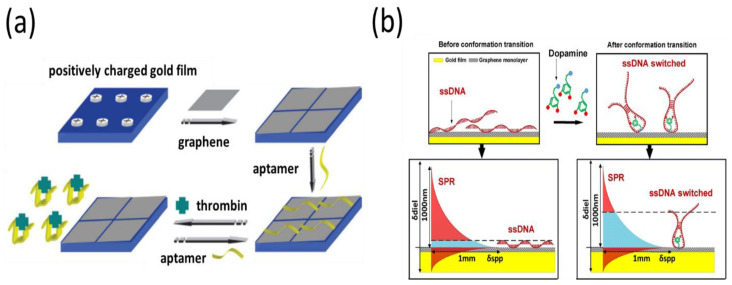
(**a**) Fabrication of the graphene-based interface with non-covalent aptamer immobilization for the detection of thrombin. (**b**) Significant plasmon phase velocity change by the conformation change of the ssDNA aptamer after the addition of dopamine molecules. Reproduced with permission from [90,91]. Royal Society of Chemistry, 2011. Elsevier B.V., 2018.

Graphene-based SPR aptasensors have been increasingly used for protein detection since their first report in 2011 [90]. Prior to detection, the SPR chip was modified to create a positively charged chip that interacted with graphene through electrostatic interactions (Figure 4a). Next, a thrombin aptamer was noncovalently adsorbed onto the graphene surface through π–π stacking interactions. Thrombin recognized and bonded to its aptamer, greatly disturbing the interaction between the aptamer and graphene. Consequently, the aptamer detached from the graphene-based SPR chip, resulting in a decreased SPR response. Based on the concept of non-covalent aptamer immobilization, Hu et al. recently developed SPR optical fiber sensors for dopamine detection using a single layer of graphene over the surface of a gold film [91]. They found that the presence of dopamine changed its aptamer conformation, which could amplify the surface refractive index signals at the fiber surface (Figure 4b). The aptasensor showed excellent sensitivity with a lower limit of detection of 10^−13^ M. The SPR sensing platform is not typically sensitive to small-molecule detection, but these studies demonstrated that the use of graphene as a sensing layer for SPR could be effective for small-molecule detection. However, a major challenge for future research is that the interaction between aptamers and the graphene-based SPR chip substantially relies on the sensing environment. In crude biological fluids such as serum, nonspecific proteins attached to the graphene surface of SPR sensors generate an unrecognized signal, limiting the detection of targets. Therefore, the accuracy and repeatability of this strategy are slightly lower than those of the covalent aptamer-modified SPR assay.

**Figure 5:**
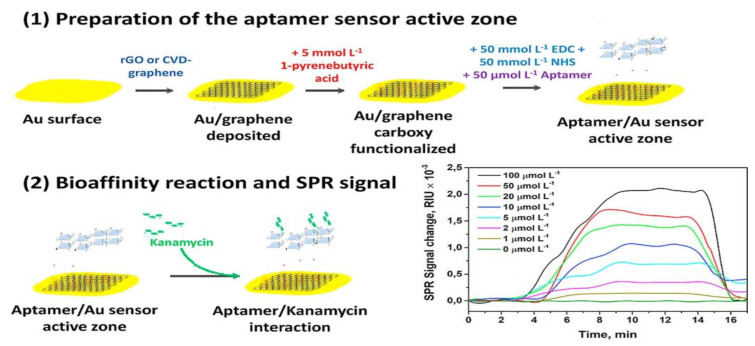
Preparation of graphene-based interface with covalent aptamer immobilization by using reduced graphene oxide and chemical vapor deposition graphene for the comparative detection of kanamycin. Reproduced with permission from [93]. Elsevier B.V., 2021.

To improve the interfacial adhesion of graphene onto a gold-coated SPR chip, Subramanian et al. fabricated a graphene matrix on gold-based SPR interfaces through the electrophoretic deposition of graphene oxide [92]. The lysozyme binding of the aptamer on the SPR interface also occurred through π-stacking interactions. The probe-label-free SPR aptasensor was reusable by immersing it in the aptamer solution, and convenient without modification of the DNA aptamer. Écija-Arenas et al. developed a graphene-modified SPR surface with covalent aptamer immobilization for the determination of kanamycin residues in foods. They used two types of graphene, reduced graphene oxide (rGO) and chemical vapor deposition (CVD) graphene, as substrate surfaces to compare the aptasensor performance (Figure 5) [93]. In their study, the CVD graphene-based gold films exhibited more homogeneous and reproducible substrates than the rGO-modified films, resulting in an optimal immobilization of aptamers. Better sensitivity of the sensor was obtained for aptasensors functionalized with CVD graphene, leading to a 7-fold enhancement in the LOD for kanamycin detection. Besides DNA aptamers, peptide aptamers are also considered as promising biorecognition probes to replace antibodies in the biosensor field [94,95]. Chiu et al. reported the peptide aptamer functionalized GO-based SPR biosensor for human chorionic gonadotropin (hCG) detection in clinical serum samples [96]. In this study, it had the lowest LOD of 1.15 pM for hCG and showed high sensitivity in the occurrence of interfering proteins.

**Figure 6:**
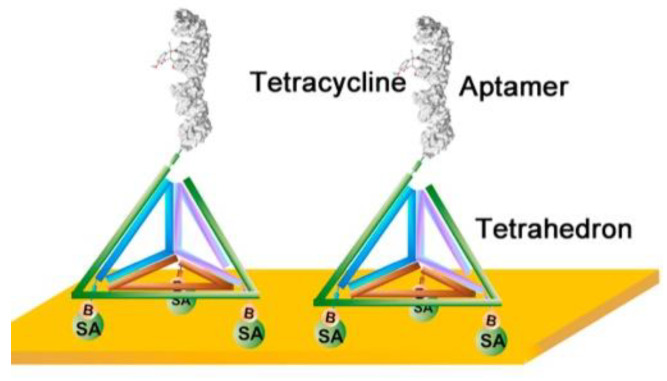
Oriented immobilization of tetracycline-binding aptamer constructed using a DNA tetrahedron nanostructure. Reproduced with permission from [97]. Elsevier B.V., 2018.

In addition to nanomaterials, Wang et al. created a novel nanostructured SPR surface with a 3D DNA nanostructure [97]. A DNA tetrahedron nanostructure was designed with a pendant aptamer probe at the top and three biotinylated sites at the base (Figure 6). This DNA tetrahedron was readily immobilized on the gold surface through biotin–avidin interaction, leaving a free-standing aptamer probe. These DNA tetrahedral nanostructures possess the unique properties of ordered orientation, well-defined probe-to-probe spacing, and structural stability. Therefore, the introduction of this 3D DNA nanostructure led to a significant increase in aptamer–tetracycline binding at the surface, which was directly translated into a remarkable increase in the signal-to-noise ratio. This is because the presence of a bulky tetrahedral structure can avoid the entanglement of the inter-aptameric probe and reduce steric hindrance effects by spatially segregating the pendant probes. Compared to the conventional anchoring approaches of aptamer probes on gold surfaces, the DNA tetrahedron structure-based SPR aptasensor exhibited a 10-fold improvement in sensitivity toward tetracycline, with a detection limit of 0.0069 μg/kg. Subsequently, the effectiveness of the sensor for tetracycline determination in several honey samples was examined, revealing acceptable recoveries in the range of 80.2% to 114.3%.

The developed sensing platforms with the one-site binding configuration are summarized in Table 1 in terms of target analytes and sensing performance. The sensitivity of SPR assays with the direct detection mode mostly falls in the nanomolar range.

## 3. Sandwich (Two-Site Binding) Sensing Mode

A pair of aptamers binding to two different epitopes in the same molecule can be easily established using a sandwich format, which is useful for developing more sensitive SPR aptasensors. One aptamer as a capture probe is immobilized on the gold surface, while the other aptamer as a reporter probe is frequently conjugated with signal-amplified tags such as nanoparticles. Although these two probes generally contain distinct oligonucleotide sequences, some dimeric proteins, such as platelet-derived growth factor-BB (PDGF-BB), have two identical binding sites [98]. Hence, in that case, the sandwich assay for PDGF-BB could be carried out using a single aptamer. However, if there is only one aptamer binding site on the target of interest, it is possible to use an antibody to construct an aptamer–antibody sandwich assay format.

### 3.1. Nanomaterial-Based Sandwich Format

Since it is possible to have limits of detection in the low nanomolar range, nanomaterial-label-based sensing strategies are extensively used in sandwich formats to amplify the SPR signal. The most promising nanomaterials employed in such formats are gold nanomaterials [99], quantum dots [100], and graphene-related materials [101], which have shown remarkable biological and chemical sensing potentials. Nanomaterial-capture probe complexes have a higher refractive index than the analytes alone, and nanomaterial-enhanced SPR sandwich aptasensors have been extensively reported [102,103,104].

**Figure 7:**
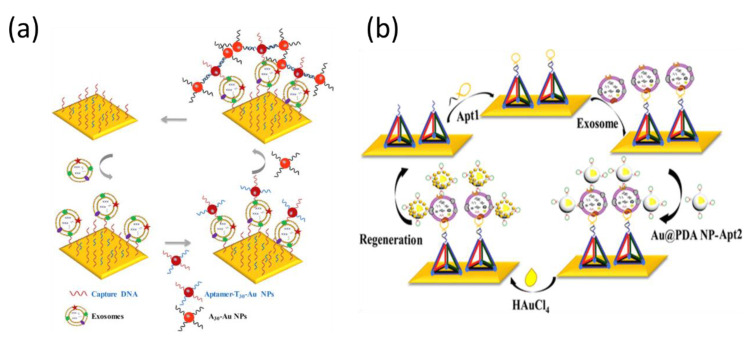
Aptamer-based SPR sensor for the determination of exosomes with (**a**) dual AuNPs and (**b**) polydopamine-functionalized AuNPs for signal amplification. Reproduced with permission from [111,112]. Elsevier B.V., 2019. Springer Nature, 2020.

With respect to gold nanomaterials, spherical gold nanoparticles (AuNPs) are the most common signal amplification tags used for many immunoassays, including lateral flow methods, colorimetric assays, and plasmonic sensing applications [105,106,107]. The optical properties of AuNPs are affected by localized SPR, which can be used to amplify SPR immunosensing. Additionally, biological ligands can be facilely linked with AuNPs, utilizing the stable chemical conjugation of mercapto and amino functional groups to gold. Therefore, AuNPs can be modified with various aptamers for the amplified detection of different analytes [108]. It is known that electronic coupling between the localized surface plasmons (SPs) of AuNPs and the SP waves associated with a gold chip can prominently amplify the SPR signal [109]. Moreover, it is noteworthy that the diameter of the AuNPs used is less than 40 nm, which is commonly used to enhance the SPR responses because the influence of scattering in AuNPs with diameters greater than 40 nm will be much stronger than the absorption [110]. Wang’s group demonstrated an efficient SPR-reliant aptasensor for breast cancer-derived exosomes with dual gold nanoparticle-assisted signal amplification (Figure 7a) [111]. First, exosomes interacted with the CD63 aptamer-immobilized gold substrate. Next, aptamer-coated T30-linked AuNPs (aptamer-T30-AuNP) were introduced to form a sandwich complex of CD63 aptamer/exosome/aptamer-T30-AuNP, resulting in a single AuNP-amplified SPR response. The A30-coated AuNPs were finally added by the hybridization of two complementary sequences (T30 and A30) to achieve dual-signal amplification. This strategy allowed an LOD of 5 ×  10^3^ exosomes/mL, providing an avenue to capture exosomes. The same group recently introduced another amplification strategy using polydopamine-functionalized AuNPs (Figure 7b) [112]. Chloroauric acid (HAuCl_4_) was reduced by polydopamine molecules to generate small AuNPs on the polydopamine-modified AuNPs, resulting in a further enhanced SPR response. The detection of exosomes with polydopamine-modified AuNPs is simpler than that of the previous method using poly(A) and T-DNA hybridization. In addition, the DNA tetrahedron-immobilized film prevented the deposition of gold on the surface during the reduction of HAuCl_4_.

**Figure 8:**
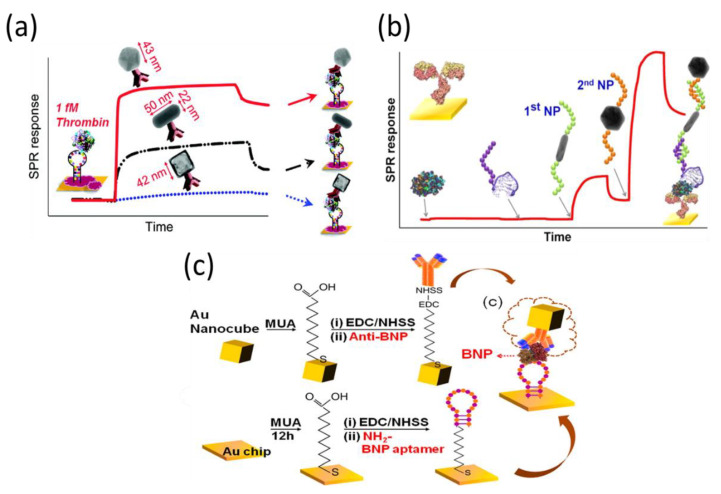
Representative SPR sensorgrams (**a**) for the sandwich detection of thrombin with anti-thrombin-coated gold nanocages, gold nanorods, and gold quasi-spherical nanoparticles, and (**b**) for the multistep amplified detection using two different nanoparticle shapes (nanorod and quasi-spherical). (**c**) Schematic overview of assay for B-type natriuretic peptide detection. Reproduced with permission from [113,114,115]. American Chemical Society, 2012 and 2014.

Recently, Lee et al. used different shapes of gold nanomaterials for the aptasensing of thrombin (Figure 8a). They found that a detection limit of 1 aM thrombin could be obtained using 40 nm quasi-spherical AuNPs, whereas detection limits of 1 fM and 10 aM were measured using gold nanocages and nanorods of the same size, respectively [113]. In another report, they developed a dual nanoparticle SPR amplification approach for detecting thrombin at concentrations as low as 0.1 aM. Two different gold nanomaterials were employed, a nanorod and a quasi-spherical nanoparticle, which resulted in a two-step SPR response amplification (Figure 8b) [114]. These methods are extremely sensitive, but their practical applications are still limited owing to their narrow detection range. Subsequently, the same group demonstrated that 50 nm gold nanocubes had a great enhancement in the SPR sensing response, similar to the quasi-spherical AuNP in the aptamer-antibody-based sandwich format (Figure 8c). Based on the combination of an aptamer-anchored gold substrate and antibody-linked gold nanocubes, the use of SPR measurements to detect B-type natriuretic peptide could be achieved down to 1 aM. A linear response range was obtained over a wide concentration range (1 aM–500 nM) [115].

Magnetic nanoparticles (MNPs) are commonly used for the separation and concentration of analytes before the detection event without the need for expensive or sophisticated equipment. For the SPR sensing applications, as MNPs do not possess the optical property of localized SPs, these particles improve the SPR performance only through their high refractive index and large mass effects, and have been successfully used in SPR analysis [116]. Chen et al. presented a highly sensitive SPR cytosensor for the detection of breast cancer cells. First, human mucin-1 (MUC1) aptamers were immobilized on the gold surface. When breast cancer cells were captured by MUC1 aptamers, folic acid-conjugated MNPs, as the second detection probe, formed a sandwich SPR assay with an LOD below 500 cells/mL [117]. The addition of a plasmonic element has been reported by coating particles with a nanoscale layer of gold, creating core–shell magnetic nanoparticles for enhanced SPR. Despite the sensing potential of MNPs, their surfaces are not fully compatible with well-defined surface chemistry, limiting their various sensing applications. Thus, the synthesis of gold-coated magnetic nanoparticles (Au@MNPs) has overcome this limitation and has been applied to SPR aptasensors [118,119]. These Au@MNPs, possessing the advantages of optical and magnetic properties and gold surface chemistry, can be highly versatile materials for the enhancement of the SPR response.

**Figure 9:**
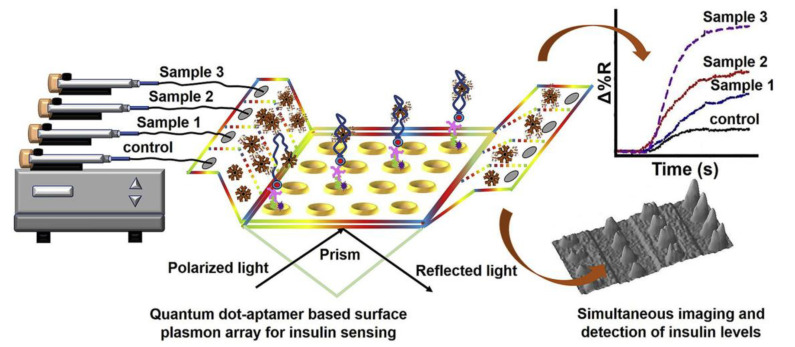
SPR microarray aptasensor for detection of serum insulin in patient samples. Reproduced with permission from [100], Elsevier B.V., 2020.

Quantum dots (QDs) have been extensively employed in biological imaging and as energy donors for use in fluorescence resonance energy transfer (FRET) sensors. Owing to their unique optical and electrical properties, the use of QDs has recently been applied to SPR biosensors to achieve high sensitivity. For example, Vance et al. used aptamer-QD conjugates for the quantitative detection of C-reactive protein (CRP) in clinical samples, with high specificity [120]. The sandwich configuration increased the SPR signal amplification 10-fold and yielded a detection limit as low as 5 fg/mL for CRP. Although the sensitivity has been significantly improved using QDs, the exact mechanism of signal improvement is still not fully understood. One hypothesis suggests that a bidirectional relationship exists between QDs and SPs. Propagating SPs interact with QDs on a metal surface and induce photon emission from QDs, and the excited QDs prompt the generation of propagating SPs [121]. Recently, another aptamer-functionalized QD-based sensor was reported by Singh [100]. An array chip incorporated with QDs was employed for the detection of insulin in serum samples of diabetic patients (Figure 9). In this study, nonspecific binding was reduced for the immobilization of high-molecular-weight dendrimers on the cysteamine layer. The designed aptasensor could detect 5 pM of serum insulin, which is important for detecting concentrations of insulin in complex clinical samples.

**Figure 10:**
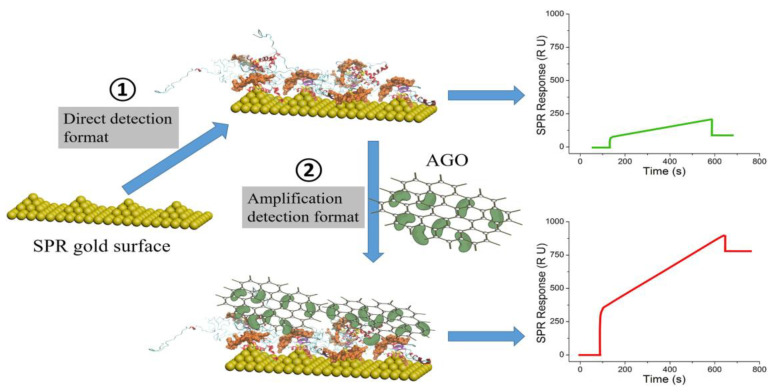
Aptamer-graphene oxide-based amplified SPR sandwich immunoassay for the quantitation of scrapie prion protein. Reproduced with permission from [122], Elsevier B.V., 2017.

In the Section 2.2, graphene-based nanomaterials were used as immobilization substrates of aptamer probes on the SPR chips. In the sandwich format, secondary aptamer-linked graphene-related materials were used for the enhancement of SPR aptasensors. Although both formats of sensors have been found to increase SPR sensitivity, there are no reports comparing the sensitivity of these two formats under the same condition. The sensing applications of graphene in the SPR sandwich configuration were reported by several research groups. Lou et al. used aptamer-modified graphene oxide (GO) sheets for enhancing SPR signals (Figure 10) [122]. The detection limit for the prion disease-associated isoform was achieved at 1 pg/mL, a 156-fold improvement over that of direct SPR detection. In addition to the above-mentioned nanomaterials, some studies have demonstrated the superior properties of hybrid nanomaterials for aptamer detection, such as polymer dots [123]. However, the use of polymer dots in SPR-based aptamer-sensing applications has not been reported and should be explored.

### 3.2. Isothermal Amplification-Based Sandwich Format

In addition to the use of nanomaterials, various isothermal amplification approaches have been explored for SPR aptasensors. In these strategies, target molecules from sample solutions are captured by immobilized antibodies or aptamers. Detection probes include the secondary aptamer region for targets and the initiation region as the primer for the isothermal amplification reaction, which are then introduced to bind to the captured target molecules. Unbound detection probes are washed away, and isothermal amplification is performed.

**Figure 11:**
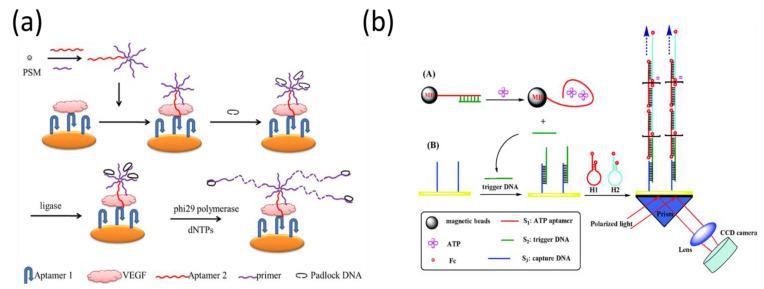
Isothermal amplification-based SPR biosensor using (**a**) enzymatic RCA and (**b**) enzyme-free HCR methods. Reproduced with permission from [124,131]. Elsevier B.V., 2014. Royal Society of Chemistry, 2014.

Rolling circle amplification (RCA) is one of the most popular isothermal amplification strategies in the SPR sandwich format, which produces a long ssDNA product by unidirectionally replicating a circular ssDNA template many times. For example, Chen et al. employed an RCA assay to improve SPR performance for the detection of vascular endothelial growth factor (Figure 11a) [124]. The detection limit was 100 pg/mL, which was slightly better than that of electrochemical methods. To notably amplify the sensitivity, He et al. developed two-step signal-amplification strategies by combining RCA and AuNPs for the detection of cancer cells and thrombin [125]. The presence of target molecules interacted with the immobilized capture probes and the detection probe with the RCA primer, resulting in the initiation of the RCA reaction. Then, RCA products were hybridized with the AuNP-modified DNA probe, producing significant amplification efficiency. In their assays, RCA-AuNPs amplified the SPR signal by almost nine orders of magnitude as compared to the direct detection, which enhanced the signal by approximately five orders of magnitude compared with the AuNP-amplified sandwich SPR-sensing configuration.

### 3.3. Other Emerging Amplification Technologies

In addition to enzyme-based isothermal amplification, recent studies have used enzyme-free reactions to amplify the reaction signal using self-assembling nucleic acids. Without the requirement of enzymes, the self-assembly of nucleic acid molecules can be triggered by free energy, and many nucleic acid byproducts can be generated through the recycling reaction. There are two common types of nucleic acid self-assembly methods, catalytic hairpin assembly (CHA) [126] and hybridization chain reaction (HCR) [127], which have been widely used in various analyses. This amplification reaction also allows for the analysis of various low concentrations of biological molecules from crude samples, such as DNA methylation and cancer biomarker detection [128,129,130]. Compared to traditional sandwich methods with the targets in the middle, these emerging amplification approaches utilized the byproducts of the target-catalyzed reaction to generate a DNA super-sandwich structure for the quantitative analysis of targets. For example, Li et al. developed a multi-step amplification scheme using an integrative approach from HCR, magnetic beads (MBs), and strand displacement for the detection of ATP (Figure 11b) [131].

**Figure 12:**
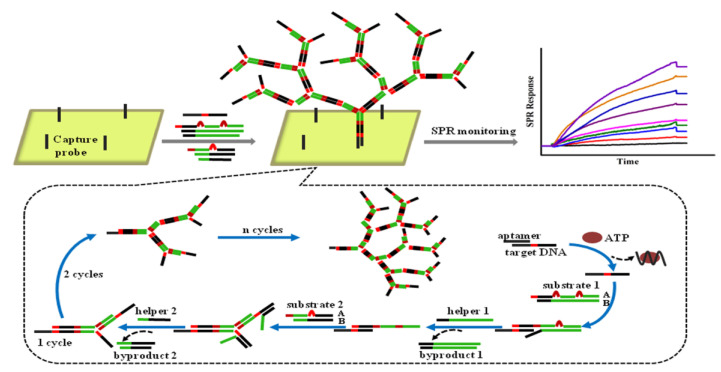
Nonlinear hybridization chain reaction-based SPR sensing strategy for the detection of ATP. Reproduced with permission from [136]. Elsevier B.V., 2017.

When ATP is introduced into the solution with aptamer-modified MBs, aptamers bind to ATP and form a complex structure, resulting in the release of complementary DNA. After magnetic separation, a solution containing complementary DNA as trigger DNA was introduced into the gold chip, and the HCR reaction was initiated by the trigger DNA. Compared with other signal-amplified SPR sensors, there are few reports based on CHA or HCR strategies for aptamer detection. This may be because the resulting nucleic acid byproducts are still small molecules that only cause a small SPR response. The efficiency of signal amplification is still not as high as that of amplification using nanomaterials such as AuNPs. To overcome this limitation, a nonlinear HCR amplification strategy was introduced, which has been used in various analysis methods [132,133,134]. In contrast to general HCR, nonlinear HCR is composed of more complex components, including a trigger DNA sequence, two dsDNA substrates with bridge loops in the middle, and two assistant DNA fragments, which can be assembled into highly branched DNA nanostructures in the presence of target proteins [135]. As a result, a non-linear HCR can achieve better amplification efficiency and larger molecular weight. Moreover, the designed DNA components for the nonlinear HCR have less secondary structure, so the amplification reaction can be completed faster. For example, Ding et al. reported real-time detection of adenosine triphosphate (ATP) using a nonlinear HCR amplification-based SPR biosensor (Figure 12) [136]. The existence of ATP can induce a nonlinear HCR allosteric effect, which leads to the dendritic growth of the DNA nanostructure on the sensing chip’s surface. Thus, the SPR response, which relies on the mass of the DNA molecules bound to the surface of the sensing chip, can be significantly increased by a nonlinear HCR amplification strategy. Concisely, under optimized conditions, the developed biosensor has demonstrated dynamic range (from 0.1 to 10 μM), and an LOD of 0.1 μM. Since the normal value of the concentration of ATP in humans is approximately 1 μM, this amplified SPR aptasensor has the potential to be used for ATP detection in humans.

The developed sensing platforms with the two-site binding configuration are summarized in Table 2 in terms of sandwich design and sensing performance. The ultrasensitive sandwich-based SPR assays in the attomolar range have been developed.

## 4. Summary and Outlook

This paper reviews state-of-the-art aptameric SPR-based biosensors and chemical sensors that function through one- and two-binding site modes. The one-binding site mode assay without labeling is a direct measurement of the target molecules, which is a simple and fast detection method. Nevertheless, the sensitivity of state-of-the-art aptamer SPR biosensors in the one-binding site mode is still insufficient to ensure a reliable non-invasive examination of low-concentration target molecules. Hence, with the aid of aptamer-modified nanomaterials as well as self-assembling nucleic acid-based nanostructures, different approaches in the two-binding-site mode have been demonstrated to enhance the sensitivity of SPR aptamer sensors. Although the integration of nanostructures and nanomaterials into the establishment of aptasensor systems has notable advantages, it increases the complexity of the sensor design, leading to higher costs, which may limit its applicability in low-resource settings. Moreover, sensor stability and manufacturing reproducibility are major challenges.

The aforementioned problems need to be solved, and thus, research efforts in the SPR field will likely concentrate on the following areas. A number of existing but underdeveloped aptamers are expected to be used in the design and construction of SPR aptamer sensors. Meanwhile, breakthroughs will also be made in further optimizing current SPR aptasensing to ensure its compatibility with real sample analysis. The availability of a cost-effective and integrated SPR aptamer biosensor system with smartphone devices could have a major positive impact on human health.

## Figures and Tables

**Table 1:** List of aptamer SPR assays with the one-site binding configuration for the detection of bio/molecules. (NR: not reported).

Basic SPR Assay						
Immobilized Aptamer Probe	Analyte		Response Time	Detection Range	Limit of Detection	Ref.
DNA	kanamycin and neomycin		10 min	0.002–0.48 μg/mL (kanamycin) 0.003–0.72 μg/mL (neomycin)	0.89 ng/mL (kanamycin) 1.55 ng/mL (neomycin)	[61]
DNA	*Pseudomonas aeruginosa*		70 min	10–10^3^ cfu/mL	10 cfu/mL	[62]
DNA	thrombin		60 min	5–20 nM	0.7 nM	[63]
DNA	Glycated hemoglobin (HbA1c)		50 min	73–294 nM	2.55 nM	[64]
DNA	*Escherichia coli* (*E. coli*) and *Staphylococcus aureus* (*S. aureus*)		80 min	10^5^–10^8^ cfu/mL 10^6^–10^8^ cfu/mL	10^5^ cfu/mL (*E. coli*) 10^6^ cfu/mL (*S. aureus*)	[65]
DNA	lysozyme		20 min	0.05–1 μg/mL	0.035 μg/mL	[66]
DNA	aflatoxin B1 (AFB1)		150 s	0.19–200 ng/mL	0.19 ng/mL	[67]
RNA	acute myeloid leukemia 1 protein (AML1)		200 s	NR	NR	[68]
DNA	Glycated hemoglobin (HbA1c)		15 min	NR	2.4 nM	[73]
DNA	thrombin		10 min	1.35–27 nM	1.35 nM	[78]
DNA	IgE		10 min	0.156–40 μM	NR	[80]
DNA	L-tyrosinamide		10 min	0.010–250 μM	10 nM	[82]
**SPR Assay Using Nanostructural Surface Design**						
**Nanostructural Surface Design**	**Aptamer Type**	**Analyte**	**Response Time**	**Detection Range**	**Limit of Detection**	**Ref.**
graphene-coated gold surface	DNA	thrombin	65 min	0.08–200 nM	0.05 nM	[90]
graphene-coated gold surface	DNA	dopamine	NR	10^−13^–10^−8^ M	1.66 × 10^−13^ M	[91]
reduced graphene oxide(rGO)-coated gold surface	DNA	lysozyme	NR	0.5–200 nM	0.5 nM	[92]
CVD-graphene- and rGO-coated gold surface	DNA	kanamycin	20 min	1–100 μM (CVD-graphene) 5.88–100 μM (rGO)	0.28 μM (CVD-graphene) 1.79 μM (rGO)	[93]
carboxyl-GO-coated gold surface	peptide	human chorionic gonadotropin (hCG)	400 s	2–100 pM	1.15 pM	[96]
DNA tetrahedron-immobilized gold surface	DNA	tetracycline	2 min	0.01–1000 μg/kg	0.0069 μg/kg	[97]

**Table 2:** List of aptamer SPR assays with the two-site binding configuration for the detection of bio/molecules. (NR: not reported).

Nanomaterial-Based Sandwich Format						
Sandwich Design (Ligand 1-Linked Nanomaterial/ Analyte/ Ligand 2 Immobilized on the Surface)		Aptamer Type	Response Time	Detection Range	Limit of Detection	Ref.
antibody-magnetic nanoparticle/ insulin/ aptamer		DNA	13 min	0.8–250 pM	0.8 pM	[100]
aptamer I-gold nanorod/ norovirus capsid protein/ aptamer II		DNA	50 min	70–500 aM	50 aM	[102]
aptamer I-dual gold nanoparticle (T30-AuNP/A30-AuNP)/ exosome/ aptamer II		DNA	60 min	NR	5 × 10^3^ particles/mL	[111]
aptamer I-polydopamine-functionalized gold nanoparticle/ exosome/ aptamer II		DNA	40 min	NR	5.6 × 10^5^ particles/mL	[112]
antibody-gold nanocage(AuNC), gold nanorod(AuNR), or gold quasi-spherical nanoparticles (AuQNP)/ thrombin/ aptamer		DNA	25 min	1 aM–1 fM (AuQNP) 10 aM–10 fM (AuNR) 1 fM–1 pM (AuNC)	1 aM (AuQNP) 10 aM (AuNR) 1 fM (AuNC)	[113]
aptamer I-dual gold nanomaterials (T20-AuNR/A30-AuQNP)/ thrombin/ aptamer II		DNA	100 min	0.1–2 aM.	0.1 aM	[114]
antibody-gold nanocube/ B-type natriuretic peptide/ aptamer		DNA	35 min	1 aM–500 nM	1 aM	[115]
folic acid-magnetic nanoparticle breast cancer cells (MCF-7)/ aptamer		DNA	333 min	5 × 10^2^–10^4^ cells/mL	5 × 10^2^ cells/mL	[117]
aptamer I-gold capped magnetic nanoparticle/ thrombin/ aptamer II		DNA	60 min	0.1–100 nM	0.1 nM	[119]
aptamer I-near-infrared quantum dot/ C-reactive protein/ aptamer II		DNA	183 min	5–5000 fg/mL	5 fg/mL	[120]
aptamer-graphene oxide/ prion disease-associated isoform/ intramolecular thiol group		DNA	40 min	4.24 × 10^−5^–4.24 × 10^−2^ nM	4.24 × 10^−5^ nM	[122]
**Isothermal Amplification-based Sandwich Format**						
**Isothermal Amplification Method**	**Sandwich Design** **(Ligand 1/** **Analyte/** **Ligand 2)**	**Aptamer** **Type**	**Response Time**	**Detection Range**	**Limit of** **Detection**	**Ref.**
rolling circle amplification (RCA)	aptamer I for the generation of RCA product/ VEGF/ aptamer II	DNA	333 min	10^−10^–10^−6^ g/mL	10^−10^ g/mL	[124]
rolling circle amplification	aptamer I-linked magnetic nanoparticle for the generation of RCA product/ romas cell/ aptamer II	DNA	NR	10–5000 cells/mL	10 cells/mL	[125]
**Other Emerging Amplification Technologies**						
**Amplification Method**	**Sandwich Design** **(Ligand 1/** **DNA byproduct/** **Ligand 2)**	**Analyte**	**Response Time**	**Detection Range**	**Limit of** **Detection**	**Ref.**
hybridization chain reaction	detection probe for the generation of linear DNA structure/ DNA byproduct/ capture probe	Adenosine triphosphate (ATP)	130 min	1–5000 nM	0.48 nM	[131]
nonlinear hybridization chain reaction	detection probe for the generation of branched DNA nanostructure/ DNA byproduct/ capture probe	ATP	67 min	1 pM–1 nM	0.85 pM	[136]
